# Effect of depth order on linear vection with optical flows

**DOI:** 10.1068/i0671

**Published:** 2014-12-01

**Authors:** Yasuhiro Seya, Takayuki Tsuji, Hiroyuki Shinoda

**Affiliations:** Department of Human and Computer Intelligence, Ritsumeikan University, Kusatsu, Shiga, Japan; e-mail: yseya@fc.ritsumei.ac.jp; Graduate School of Information Science and Engineering, Ritsumeikan University, Kusatsu, Shiga, Japan; e-mail: is026089@ed.ritsumei.ac.jp; Department of Human and Computer Intelligence, Ritsumeikan University, Kusatsu, Shiga, Japan; e-mail: hshinoda@is.ritsumei.ac.jp

**Keywords:** linear vection, binocular disparity, three-dimensional space

## Abstract

In the present study, the effects of depth order on forward and backward vection were examined using optical flows simulating motion in depth (i.e., approaching or receding). In an experiment, space extending 10 or 20 m in depth was simulated, and the space was divided into foreground and background spaces. In each space, a random-dot pattern was presented and the binocular disparity, size, and velocity of each dot were continuously manipulated in a way consistent with the depth being simulated. Participants reported whether they perceived vection. Latency, total duration (i.e., the amount of time that participants reported perceiving vection during a 60-s presentation), and strong-vection duration (i.e., the amount of time that participants reported perceiving strong vection) were measured. The results indicated that, even though the dots making up the optical flow were much smaller and slower moving in the background space than in the foreground space, vection was strongly dependent on flow motion in the background space. This supports the idea that the perceptual system uses background stimulus motion as a reliable cue for self-motion perception.

## 1 Introduction

When observers view a moving stimulus in a large area of their visual field, they perceive self-motion in the opposite direction of the stimulus motion. This phenomenon is called vection (Brandt, Dichgans, & Koenig, 1973), and is considered to be evidence that visual information induces self-motion perception independently of actual body movements (for a review, see Warren, 1995). When an observer actually moves (or rotates his or her head horizontally), self-motion perception is rapidly created by vestibular information from vestibular organs. However, because vestibular organs respond only to the acceleration of body movements, it is reasonable to assume that visual information of counter-motion of the visual scene causes sustained perception of self-motion. This notion is supported by physiological evidence indicating that visual inputs (such as optical flow caused by an observer's locomotion), as well as vestibular information, activate the vestibular nuclei (e.g., Dichgans, Schmidt, & Graf, 1973; Henn, Young, & Finley, 1974; Hoffmann & Distler, 1986; for reviews, see Barmack, 2003; Ilg, 1997).

Many studies have revealed visual conditions and/or stimulus attributes that systematically change the strength and/or direction of vection. For example, the strength of vection increases with increasing stimulus velocity (Brandt et al., 1973; Nakamura & Shimojo, 1999) and size (Brandt et al., 1973; Berthoz, Pavard, & Young, 1975; Held, Dichgans, & Bauer, 1975; see also Leibowitz, Post, Rodemer, Wadlington, & Lundy, 1980), and the direction of vection has been shown to be modulated by attention (Kitazaki & Sato, 2003) such that the direction of vection is determined by nonattended motion.

The order of stimuli in depth is well known to affect vection. Many studies have reported that if visual stimuli differ in depth, vection is determined by the more distant stimulus (Brandt, Wist, & Dichgans, 1975; Delmore & Martin, 1986; Howard & Howard, 1994; Ito & Shibata, 2005; Ohmi, Howard, & Landolt, 1987; Ohmi & Howard, 1988; Seno, Ito, & Sunaga, 2009). For example, Brandt et al. (1975) showed that circular vection (rotation vection) was markedly reduced when stationary bars were presented behind a moving display, while vection was not affected when the bars were presented in front of the display. Ohmi et al. (1987) reported that circular vection was determined by the distant stimulus or the stimulus perceived to be distant. Interestingly, they found that vection depended on the distant display even when the close and distant stimuli were moving in opposite directions (see also Howard & Heckmann, 1989).

Although the reason why vection depends on depth order is still controversial, an ecological theory has been proposed (see Nakamura & Shimojo, 1999). In the real world, the part of the scene that is perceived as more distant (i.e., the background) rarely moves relative to the observer's body unless the observer moves, so retinal motion of the background would likely correspond to the observer's self-motion (Gibson, 1979). In contrast, objects close to the observer (i.e., the foreground) generally move irrespective of the observer's motion. Therefore, the perceptual system may use the background, rather than the foreground, as a reliable cue for self-motion perception. It should be noted that this theory does not mean that the foreground has no effect on vection. Indeed, several studies have shown that the foreground enhances vection strength (Howard & Howard, 1994; Nakamura & Shimojo, 1999). Furthermore, this idea cannot explain some cases where the foreground determines the strength and/or direction of vection (e.g., inverted vection; Nakamura, 2004; Nakamura & Shimojo, 2000; Nakamura & Shimojo, 2003, see also Howard & Heckmann, 1989).

As we reviewed above, many studies have reported that vection is dependent on depth order. However, most previous studies examined this relationship by using rotating or translating (in front of the observers) stimuli, and, to our knowledge, research on this topic using stimuli moving in depth, i.e., approaching or receding, is scarce. Several studies have examined the effects of depth order on vection by using an expanding or contracting stimulus, which simulates an approaching or receding stimulus and is known to induce linear vection in depth (Andersen, 1986; Ohmi & Howard, 1988; Telford & Frost 1993). Ohmi and Howard (1988) examined forward vection when a radially expanding pattern of dots was presented. In their study, binocular disparity of stationary dots was manipulated to make them appear superimposed in front of or behind expanding dots. The results showed that forward vection was markedly reduced when the stationary dots were presented behind the expanding display, while it did not change much when the stationary dots were presented in front of the display. By simultaneously presenting expanding and contracting flows overlapped with different disparities, Ito and Shibata (2005) examined the effect of depth order on vection. The results indicated that the vection direction was determined by the flow that was perceived to be more distant, e.g., when contracting flow was perceived to be more distant than expanding flow, participants reported backward vection.

In the present study, we further examined effects of background on linear vection by using random-dot patterns simulating approaching or receding dots. The motivation for our study was twofold. First, we wanted to examine vection by using a more ecologically valid stimulus (Palmisano, 1996, 2002; Palmisano & Chan, 2004). In real-life situations, an observer's locomotion, such as when he or she walks or drives a car, causes optical flow to approach (or recede) along the line of sight. Assuming that the perceptual system is adapted in such a way that the background is used as a reliable cue for self-motion perception, one would expect that strong dependency would be observed when approaching or receding stimuli are used. By “ecologically valid stimulus,” we mean that the stimulus has features, e.g., optical flows and depth cues (binocular disparity, objects' size and velocity), which are utilized for perceptual activities in real-life situations. Therefore, we do not mean that the stimulus is realistic. Second, we wanted to examine the effects of depth order on vection by continuously manipulating the dot size, velocity, and disparity in a way consistent with the depth being simulated, not usually manipulated in previous studies (although Ohmi & Howard, 1988, manipulated velocity of dots according to the depth simulated). In a natural scene, when an observer moves, the retinal images of distant objects are smaller and move more slowly than those of near objects. If we assume that the perceptual system is adapted to such visual environments, it is likely that when different depth objects are identical in size and velocity on the retina, distant objects are perceived to be larger and faster than near ones. As a result, perceptual (subjective) stimulus intensity could be higher for the distant objects than near ones, which might cause the dependency of vection on the background. Furthermore, in previous studies, effects of depth order were examined by using optical flow patterns presented on two-dimensional planes (in different depths), not those with a three-dimensional volume. It is, therefore, possible that depth order effects would be specific to situations where depth cues were limited.

In the present study, relatively large spaces (i.e., 10 m and 20 m) were simulated, and each space was divided into foreground and background spaces. We selected these large spaces, because the retinal image size and velocity of dots in the background space were sufficiently smaller and slower than (but perceptual size and velocity were equal to) those in the foreground space. As we mentioned above, it is possible that the location of the observer's attention affects vection, despite the fact that Kitazaki and Sato (2003) reported that attentional effects on vection were overridden when stimuli had a depth order. Nonetheless, to examine this possibility, we manipulated the location of the fixation point. During the task, participants were presented with random-dot patterns approaching or receding in each space. Latency to the onset of vection and amount of time during which participants perceived vection were measured, as in previous studies (e.g., Berthoz et al., 1975; Nakamura & Shimojo, 1999). Strength of vection (i.e., strong vs. weak) was also measured.

## 2 Method

### 2.1 Participants

Participants were 10 participants (nine males and one female; mean age of 22.9 in the range of 22–24). One of them was the second author and the others were naïve to the purpose of the experiment. Participants provided written informed consent to the experiment before participation. All of them had normal or corrected-to-normal vision.

### 2.2 Apparatus and stimuli

A personal computer (Apple Mac Pro Early 2009) with a graphics board (NVIDIA Quadro4000) was used to control the experiment and generate stimuli that were rear-projected onto a screen with a 3D projector (Vivitek D795WT) with a refresh rate of 120 Hz. The size of the screen area in which the stimuli were presented was 95 cm (horizontal) × 95 cm (vertical), subtending 72.3° × 72.3° of visual angle. For a stereoscopic view, stimuli were viewed binocularly with goggles (BenQ 3DGS-01) from a distance of 65 cm. The experimental program was written with MATLAB with Psychophysics Toolbox extensions (Brainard, 1997; Pelli, 1997).

**Figure 1.**
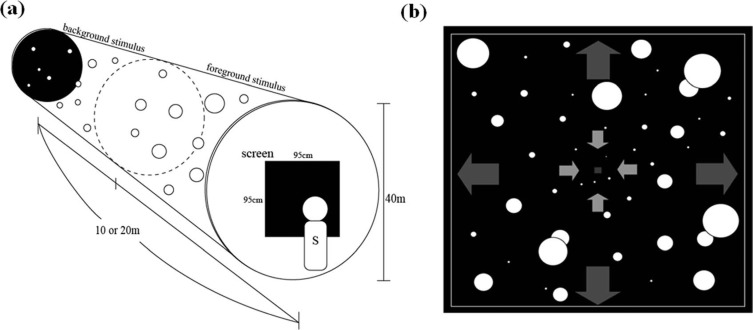
Illustration of the stimulus display used in the present study. (a) In the simulated cylindrical space, dots moved in depth. (b) Dots in the foreground and background spaces, a fixation stimulus, and a frame were presented in the display.

Figure 1 illustrates the stimulus display used in the present study. We simulated a cylindrical space spreading in depth (Figure 1a). The size of the simulated space was either 10 m or 20 m in depth with a diameter of 40 m. The space was divided into background and foreground spaces. In each space, a random-dot pattern was presented in white (luminance 35.2 cd/m^2^) on a black background (luminance 0.31 cd/m^2^). The position of each dot was refreshed at a rate of 60 Hz and each dot was stationary (i.e., 0 km/h) or was simulated to move in depth (approaching or receding) by manipulating uncrossed disparity depending on its distance from the observer. Thus, there were four combinations of the foreground and background flows: (1) stationary foreground and moving background, (2) moving foreground and stationary background, (3) moving foreground and background in the same direction, and (4) moving foreground and background in opposite directions. A square frame was presented in white and surrounded the screen area in which stimuli were presented (see Figure 1b). This frame served as a window through which the observer could see outside. The maximum uncrossed disparity was 5.53°. It should be noted that when dots moved in a space between the screen and the observer, a crossed disparity was added to them (the size of the disparity was changed depending on the distance from the observer). In each space, the dots disappeared when they reached the edge of the space and reappeared at the opposite side. The simulated velocity of dots was either 5 km/h or 10 km/h. The simulated dot size was 4 cm in diameter and the retinal size of dot changed depending on distance from the observer. In the 10-m condition, a total of 500 dots were presented (250 dots in each space), and in the 20-m condition a total of 1,000 dots were presented (500 dots in each space).

In the display, a 1 × 1 cm red fixation dot (luminance 3.93 cd/m^2^) was presented at the center of the screen. The fixation dot was presented in the middle of either the background or foreground space.

### 2.3 Procedure

The experiment was conducted in a dark booth. Participants were seated comfortably with their heads upright. No apparatus was used to support head position, and participants maintained their posture and head position by themselves during the experimental sessions. After 3 min of dark adaptation, the experimental session began. At the beginning of each trial, stationary dots in the background (i.e., background flow) and the foreground (i.e., foreground flow) spaces and the fixation were presented until participants pressed the enter key to begin the trial. After the key-press, the dots moved (or remained stationary) for 60 s. The dots and fixation then disappeared. During the presentation of stimulus motion, participants were asked to keep their eyes on the fixation and to press one of four labeled keys on a keypad when they experienced vection. The four keys corresponded to the subjective strength (strong or weak) and the direction (forward or backward) of vection. Participants were asked to keep the button depressed whenever they experienced vection. Latency and duration of key presses were measured.

There were three trials for each combination of space size (10 m and 20 m), fixation position (foreground and background), foreground velocity (0 km/h, ±5 km/h, and ±10 km/h), and background velocity (0 km/h, ±5 km/h, and ±10 km/h). Thus, there were 300 trials in total. There were six sessions of 50 trials. In each session, the size of the space was fixed and the other conditions were randomized across trials. Five of the 10 participants performed the 10-m condition first, and the other five participants completed the 20-m condition first. All participants completed conditions over three or six days, depending on their schedule and availability each day. There were 30-min rest periods between sessions. Before the main experiment, all participants practiced the task for an hour. During the practice, they were asked to determine the criterion of strong vection.

### 2.4 Data analysis

In the present study, we analyzed three measures: latency, total duration, and strong-vection duration. Latency was defined as the time from the initiation of flow motion to the participant first pressing one of four buttons. In trials where no vection was reported, we assigned a value of 60 s to the latency, which was equal to the whole duration of optical-flow stimulation as in previous studies (e.g., Andersen & Braunstein, 1985; Palmisano, Burke, & Allison, 2003; Telford, Spratley, & Frost, 1992). Total duration was calculated as the total time of weak and strong vections indicated by participants. Strong-vection duration was calculated as the time of strong vection. Although we measured each duration of forward and backward vection for each condition, five participants consistently reported perceiving forward vection when the background dots were approaching, and backward vection when the background dots were receding. The remaining five participants reported both directions of vection in a few trials (about 4% of the total trials on average). However, when the background dots were approaching, the duration of forward vection was always longer than that of backward vection. In contrast, when the background dots were receding, the duration of backward vection was always longer than that of forward vection. For these participants, we used the longer of the two vections as data in the analysis.

## 3 Results

**Figure 2.**
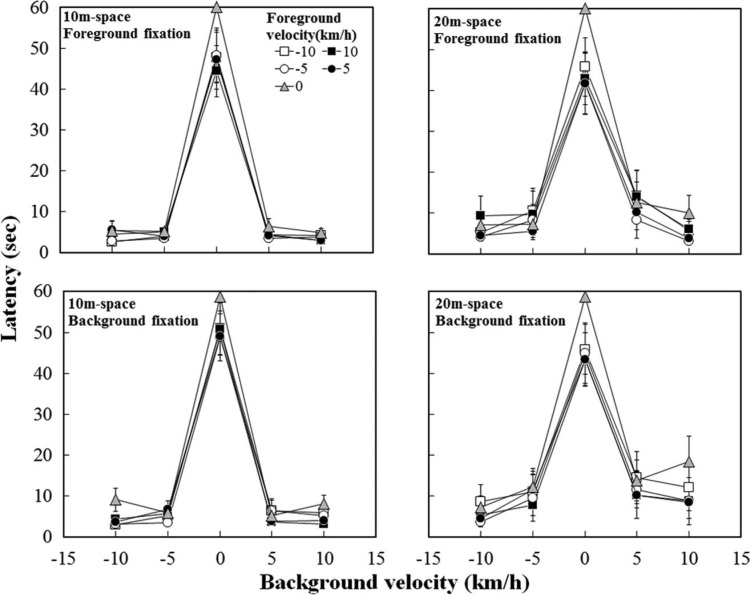
Mean latency as a function of background flow velocity, with a separate line for each foreground flow velocity. Vertical bars indicate standard errors of the mean.

Figure 2 shows the mean latency as a function of background velocity for each foreground velocity under the 10-m (left panels) and 20-m (right panels) conditions. The upper panels show results under the foreground-fixation condition where the fixation dot was presented in the middle of the foreground space, and the lower panels pertain to the background-fixation condition where the fixation dot was presented in the middle of the background space. Positive values on the horizontal axis mean that the background dots were approaching, and negative values mean that the background dots were receding. As can be seen in the figure, latencies were shorter when the background dots were in motion than when they were stationary. The latencies also appeared to be shorter when the foreground dots were in motion than when they were stationary.

A 2 (space: 10 and 20 m) × 2 (fixation: foreground and background) × 5 (foreground: 0, ± 5, ±10 km/h) × 5 (background: 0, ±5, ±10 km/h) repeated measures ANOVA was performed. The main effect of background was significant, *F* (4, 36) = 85.47, *p* < .001, verifying the dependency of latency on the background motion. The main effect of foreground was also significant, *F* (4, 36) = 7.08, *p* < .001. No other main effect was significant: space, *F* (1, 9) = 2.02, fixation, *F* (1, 9) = 3.61. There was only a significant interaction of space × background, *F* (4, 36) = 3.65, *p* = .014. A posteriori analyses with Turkey's HSD test for the effect of background revealed significantly shorter latencies when the background dots were in motion than when they were stationary (all *p*s <.05). A posteriori analyses for the effect of foreground revealed significantly shorter latencies when the foreground dots were in motion than when they were stationary (all *p*s < .05). Subsequent analysis of the interaction of space × background showed a significantly shorter latency in the 10-m than in the 20-m spaces at 5-km/h background velocity, *F* (1, 45) = 7.01, *p* = .011.

**Figure 3.**
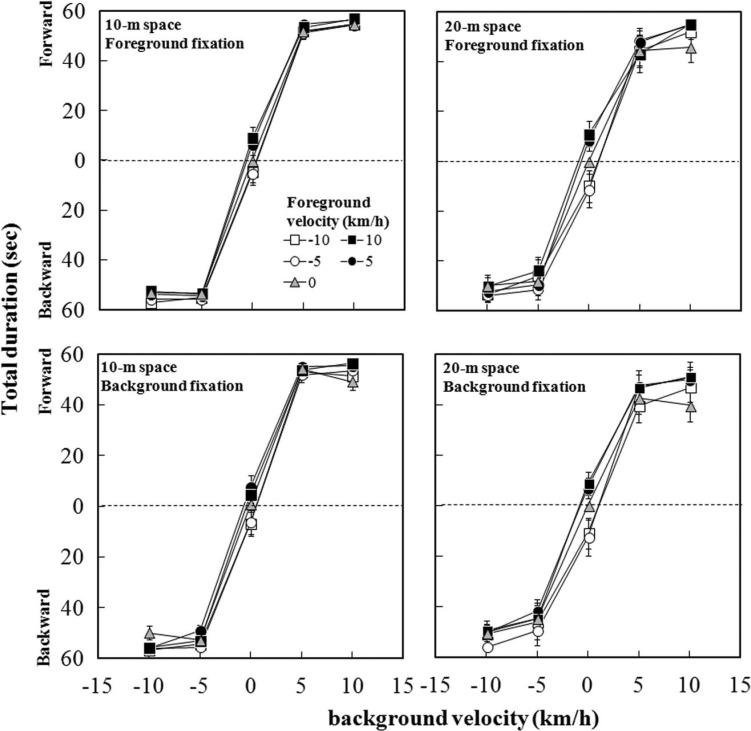
Mean total duration as a function of background flow velocity, with a separate line for each foreground flow velocity. Vertical bars indicate standard errors of the mean.

Figure 3 shows the mean total duration as a function of background velocity for each foreground velocity under the 10-m (left panels) and 20-m (right panels) conditions. The upper panels are for the foreground-fixation condition and the lower panels are for the background-fixation condition. As can readily be seen in the figure, the total duration of vection depended on the direction of background motion. When background dots were approaching, the total duration of forward vection almost reached the maximum, indicating that participants perceived forward vection as soon as the background dots started to move. Similarly, when background dots were receding, the total duration of backward vection almost reached the maximum.

A four-way repeated measures ANOVA revealed significant main effects of background, *F* (4, 36) = 116.92, *p* < .001, and foreground, *F* (4, 36) = 3.64, *p* = .014, but no significant main effect of space, *F* (1, 9) = 3.34, or fixation, *F* (1, 9) = 2.59. There were significant interactions of space × background, *F* (4, 36) = 5.29, *p* = .002, and fixation × background, *F* (4, 36) = 3.23, *p* = .023. A posteriori analyses for the effect of background showed significantly longer total durations when the background dots were moving than when they were stationary (all *p*s < .05). A posteriori analyses for the effect of foreground showed a significantly longer total duration when the foreground dots were moving at −5 km/h than when they were stationary (*p* < .05). Subsequent analysis of the space × background interaction showed a significantly longer total duration in the 10-m space than in the 20-m space at background velocities of −5 km/h, *F* (1, 45) = 7.06, *p* = .011, and 5 km/h, *F* (1, 45) = 8.52, *p* = .006. Subsequent analysis of the fixation × background interaction showed that total duration was significantly longer under the foreground-fixation condition than under the background-fixation condition at a background velocity of 10 km/h, *F* (1, 45) = 10.82, *p* = .002. A difference between the two spaces just failed to reach significance at a background velocity of −5 km/h, *F* (1, 45) = 3.82, *p* = .057.

**Figure 4.**
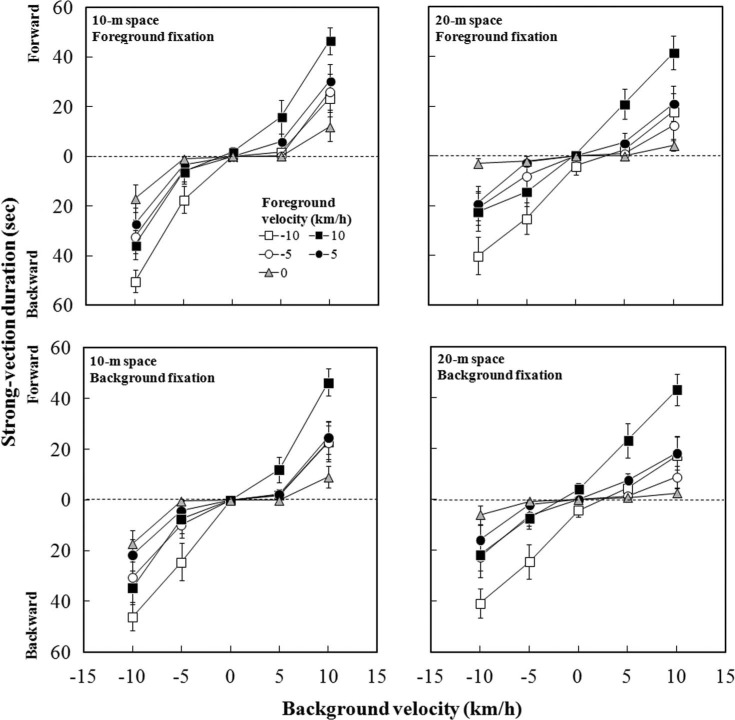
Mean duration of strong vection as a function of background flow velocity, with a separate line for each foreground flow velocity. Vertical bars indicate standard errors of the mean.

Figure 4 shows the mean duration of strong vection as a function of background velocity for each foreground velocity under the 10-m (left panels) and 20-m (right panels) conditions. The upper panels pertain to the foreground-fixation condition and the lower panels to the background-fixation condition. It is obvious that the duration of strong vection increased with increasing background velocity. It should be noted that effects of background velocity were modulated by the foreground velocity; the strong-vection durations appeared to be greater when the foreground dots moved in the same direction as the background dots rather than in the opposite direction.

A four-way repeated measures ANOVA revealed significant main effects of background, *F* (4, 36) = 38.50, *p* < .001, and foreground, *F* (4, 36) = 13.50, *p* < .001, but no significant main effect of space, *F* (1, 9) = 4.44, or fixation, *F* (1, 9) = 0.54. There were significant interactions of foreground × background, *F* (16, 144) = 4.59, *p* < .001, space × background, *F* (4, 36) = 9.33, *p* < .001, and space × fixation × background, *F* (4, 36) = 2.95, *p* = .033. A posteriori analyses for the effect of background showed significantly longer durations of strong vection at the 10-km/h and −10-km/h velocities than at the other velocities (all *p*s <.05). Strong-vection duration was also significantly longer at the −5-km/h velocity than at the 0-km/h velocity (*p* < .05). A posteriori analyses for the effect of foreground showed significantly longer durations of strong vection at the 10-km/h and −10-km/h velocities than at the other velocities (all *p*s < .05).

Subsequent analysis of the foreground × background interaction showed significant simple main effects of background at all velocities of foreground flow except for at 0 km/h [10 km/h, *F* (4, 180) = 20.72; 5 km/h, *F* (4, 180) = 8.63; −10 km/h, *F* (4, 180) = 22.14; −5 km/h, *F* (4, 180) = 9.44, all *p*s < .001]. A posteriori analyses showed a tendency of longer durations of strong vection when both the foreground and the background flows moved in the same direction than when they did in opposite directions. Under the 10-km/h condition of foreground flow, strong-vection durations were significantly longer at the 10-km/h velocity of background flow than at the other velocities (all *p*s < .05). They were significantly longer at the 5-km/h velocity than at the 0-km/h velocity (*p* < .05). They were also significantly longer at the −10-km/h velocity than at the −5-km/h and 0-km/h velocities (both *p*s < .05). Under the 5-km/h condition of foreground flow, strong-vection durations were significantly longer at the 10-km/h velocity of background flow than at the 5-km/h, 0-km/h, and −5-km/h velocities (all *p*s < .05). They were also significantly longer at the −10-km/h velocity of background flow than at the 5-km/h, 0-km/h, and −5 km/h velocities (all *p*s < .05). Under the −10-km/h condition of foreground flow, strong-vection durations were significantly longer at the −10-km/h velocity of background flow than at the other velocities (all *p*s < .05). They were also significantly longer at the −5-km/h and 10-km/h velocities than at the 0-km/h and 5-km/h velocities (all *p*s < .05). Under the −5-km/h condition of foreground, strong-vection durations were significantly longer at the −10-km/h velocity than at the 5-km/h, 0-km/h, and −5-km/h velocities (all *p*s < .05). They were also significantly longer at the 10-km/h velocity than at the 5-km/h and 0-km/h velocities (both *p*s < .05). Subsequent analysis of the space × background interaction showed significantly longer durations of strong vection in the 10-m space than in the 20-m space at the 10-km/h, *F* (1, 45) = 13.48, and −10-km/h background flow velocities, *F* (1, 45) = 22.87, both *p*s < .001.

## 4 Discussion

In the present study, we continuously manipulated dot velocity, size, and disparity corresponding to simulated distances from the observer, to examine the effects of depth order on forward and backward vection. The results showed that the latency to the onset of vection was clearly less when the background dots were in motion than when they were stationary. The results of total duration and strong-vection duration clearly showed a dependency of vection direction on the background motion; for example, participants reported perceiving forward vection when the simulated motion of the background dots was approaching. Although the latencies changed little with background velocity and the total duration almost reached the maximum when the background dots were in motion, the duration of strong vection was clearly greater when the velocity of the background dots was greater, indicating that the strength of vection increased as the background velocity increased (Nakamura & Shimojo, 1999). Taken together, these results clearly showed dependencies of vection strength and direction on the background motion. As we described in the Introduction, most studies have examined the effect of depth order on circular vection or linear vection induced by stimuli moving in two dimensions (e.g., Brandt et al., 1975; Delmore & Martin, 1986; Ohmi et al., 1987; Seno et al., 2009). A few studies have examined the effect of depth order on vection using optical flows simulating motion in three dimensions (Ito & Shibata, 2005; Ohmi & Howard, 1988). However, to our knowledge, no study has examined the effect of depth order by continuously manipulating the size, velocity, and disparity of dots according to the distance simulated. Therefore, this is the first study to report effects of depth order on depth linear vection with ecologically valid stimuli.

It should be noted that large spaces were simulated in the present study (i.e., 10 m and 20 m) and as a result the retinal images of background dots were much smaller than those of the foreground dots, and moved much slower. Nevertheless, vection was strongly dependent on the background dot motion although under some conditions there were significant differences between the 10-m and 20-m levels of space in all measures. Considering the findings of previous studies using stimuli with fixed size and velocity, these results suggest that, regardless of subjective stimulus intensity, the perceptual system uses background motion as a reliable cue for self-motion perception (e.g., Nakamura & Shimojo, 1999).

The present results of long latencies and short total and strong-vection durations in the presence of stationary background dots are in agreement with the findings of Ohmi and Howard (1988). However, the present results also showed these features when a stationary foreground was presented. This is not consistent with the results of Ohmi and Howard, who found no change in the vection strength when a stationary foreground was presented in front of a moving background. Our latter findings suggest that the foreground can influence the strength of vection, and support the notion that the foreground, in addition to the background, plays an important role in the perception of self-motion (e.g., Howard & Howard, 1994; Nakamura & Shimojo, 1999).

The discrepancy between this study and that of Ohmi and Howard (1988) might be explained by the flow patterns used. In the present study, the dots changed their size depending on the distance from the observer, whereas this was not the case in the study of Ohmi and Howard. As we mentioned in the Introduction, when objects at different depths appear identical in size on the retina, the perceptual stimulus intensity could be larger for the more distant objects than for the nearer ones. Therefore, in the work of Ohmi & Howard, the background dots might have had enough perceptual strength to induce vection consistently, regardless of the foreground dots.

The inhibitory effects of a stationary foreground on vection observed here are also inconsistent with the findings of Howard and Howard (1994) and Nakamura and Shimojo (1999). Howard and Howard hypothesized that a stationary foreground enhances vection induced by a background compared with vection induced by a single pattern (i.e., only a background is presented) because relative motion signals between the foreground and background stimuli are produced. Their results supported this hypothesis. Nakamura and Shimojo extended these findings by showing that the strength of vection was enhanced when the foreground was stationary or moved slowly in the opposite direction to a background, while it was inhibited when the foreground moved slowly along with, or fast opposite to, the background. These results clearly suggest that relative motion signals affect vection, but the relationship between relative motion signals and vection is complex (see also Nakamura & Shimojo, 2000). According to the relative motion hypothesis, vection should be larger when the foreground is stationary or moves in the opposite direction to the background and smaller when the foreground moves in the same direction as the background. In the present study, unlike Howard and Howard and Nakamura and Shimojo, we did not include control conditions under which the background dots were presented alone, so enhancement and/or reduction effects on vection due to the presence of the foreground are not clear from our data. However, the strong-vection duration results suggest that vection was significantly weaker (strong-vection duration became less) when the foreground dots moved in the opposite direction to the background than when they moved in the same direction (Figure 4). This finding, together with the finding of weak vection in the presence of a stationary foreground, is not consistent with predictions based on the relative motion hypothesis.

One may argue that the present results may be explained by the vector summation hypothesis, which suggests that vections induced by foreground and background stimuli are summated, or alternatively, that the summed foreground and background motion signals cause vection (see Nakamura & Shimojo, 2000). According to the vector summation hypothesis, one would expect no perception of vection when the foreground and background stimuli move in opposite directions at the same velocity. In addition, vection should be moderately inhibited when the foreground is stationary. However, the present results of strong vection showed a clear decrease when the foreground was stationary, and moderate levels when the foreground moved in the direction opposite to that of the background. Furthermore, even when the foreground dots moved in the direction opposite to that of the background dots, strong-vection durations were always longer when the foreground dots moved faster. These results are not consistent with the predictions of the vector summation hypothesis. Therefore, vector summation cannot account for the present results.

Under most conditions, the latency, total duration, and duration of strong vection were not influenced by whether the fixation dot was located in the foreground or background space. If we assume that participants' attention was allocated around the fixation dot, this suggests that vection is not modulated by attention. This is consistent with the findings of Kitazaki and Sato (2003), who showed that attentional effects on vection were overridden when stimuli had a depth order. One may argue that the present results did not align with the findings of studies measuring postural sway, which is known to be related to vection (e.g., Previc, 1992; Guerraz & Bronstein, 2008), in response to lateral visual motion (Bronstein & Buckwell, 1997; Guerraz & Bronstein, 2008; Guerraz, Gianna, Burchill, Gresty, & Bronstein, 2001; Meyer, Shao, Hopkins, & Robotham, 2013). In those studies, modulations of postural sway by a fixation location were reported: fixation on a stationary object within a moving background induced postural sway in the direction of background motion, while fixation on a stationary object in front of a moving background did it in the opposite direction. However, Guerraz and Bronstein (2008), who measured vection as well as postural sway, reported that vection was always induced in the direction opposite to the background motion, irrespective of the direction of postural sway. They also reported that the postural sway in the opposite direction was transient and followed by the postural sway in the direction of background motion (see also Meyer et al., 2013). These results suggest that effects of fixation location would be specific to mechanisms of postural response initiation and that they would not affect vection. Note that no significant modulations were found when background stimuli moved in depth (Meyer et al., 2013).

Two points are noteworthy concerning the methodologies used in the present study. One is that, because we employed a relatively short viewing distance (i.e., 65 cm), accommodation as a depth cue would have indicated that the stimuli were two-dimensional while the large disparity of stimuli used in the present study indicated that they were three-dimensional. As a result, information provided by accommodation and stimulus disparity may have reduced vection in the present study, on the basis that inconsistent depth information reduces vection (Palmisano, 2002). However, the results showed that the stimuli used in the present study induced vection rapidly after stimulus onset and that the total duration of vection was almost maximum when the stimuli were in motion (Figures 2 and 3). This suggests that the stimuli used in the present study sufficiently induced vection. Furthermore, because the viewing distance was fixed, these factors did not differ between the conditions. Therefore, accommodation and/or large disparity would not account for the present findings. The other point is that we did not conduct the control condition under which the background dots were presented alone, as we mentioned earlier. Although the comparison in the results between the moving and the stationary foreground conditions suggests that the stationary foreground inhibited vection induced by the background motion, the control condition should have been included for a better understanding of the effects of foreground motion on vection. In future studies, this point, as well as the effects of accommodation and disparity, needs to be examined.

In conclusion, the present study clearly demonstrated that depth linear vection is determined by the background. This is consistent with previous studies examining circular vection and vection induced by stimuli moving in two dimensions (e.g., Brandt et al., 1975; Delmore & Martin, 1986; Ohmi et al., 1987; Seno et al., 2009). The present results support the notion (e.g., Nakamura & Shimojo, 1999) that the perceptual system uses background motion, rather than foreground motion, as a reliable cue for self-motion perception.
